# SNP eQTL status and eQTL density in the adjacent region of the SNP are associated with its statistical significance in GWA studies

**DOI:** 10.1186/s12863-019-0786-0

**Published:** 2019-11-12

**Authors:** Ivan Gorlov, Xiangjun Xiao, Maureen Mayes, Olga Gorlova, Christopher Amos

**Affiliations:** 1 The Geisel School of Medicine, Department of Biomedical Data Science, Dartmouth College, HB7936, One Medical Center Dr., Dartmouth-Hitchcock Medical Center, Lebanon, NH 03756 USA; 2 Department of Medicine, Baylor College of Medicine, One Baylor Plaza, Houston, TX 77030 USA; 3 Department of Internal Medicine, Division of Rheumatology, University of Texas McGovern Medical School, Houston, TX USA

**Keywords:** Genome wide association studies (GWASs), Expression quantitative trait loci (eQTL), Statistical significance, Cancer, Gene expression

## Abstract

**Background:**

Over the relatively short history of Genome Wide Association Studies (GWASs), hundreds of GWASs have been published and thousands of disease risk-associated SNPs have been identified. Summary statistics from the conducted GWASs are often available and can be used to identify SNP features associated with the level of GWAS statistical significance. Those features could be used to select SNPs from gray zones (SNPs that are nominally significant but do not reach the genome-wide level of significance) for targeted analyses.

**Methods:**

We used summary statistics from recently published breast and lung cancer and scleroderma GWASs to explore the association between the level of the GWAS statistical significance and the expression quantitative trait loci (eQTL) status of the SNP. Data from the Genotype-Tissue Expression Project (GTEx) were used to identify eQTL SNPs.

**Results:**

We found that SNPs reported as eQTLs were more significant in GWAS (higher -log_10_p) regardless of the tissue specificity of the eQTL. Pan-tissue eQTLs (those reported as eQTLs in multiple tissues) tended to be more significant in the GWAS compared to those reported as eQTL in only one tissue type. eQTL density in the ±5 kb adjacent region of a given SNP was also positively associated with the level of GWAS statistical significance regardless of the eQTL status of the SNP. We found that SNPs located in the regions of high eQTL density were more likely to be located in regulatory elements (transcription factor or miRNA binding sites).

When SNPs were stratified by the level of statistical significance, the proportion of eQTLs was positively associated with the mean level of statistical significance in the group. The association curve reaches a plateau around -log_10_p ≈ 5. The observed associations suggest that quasi-significant SNPs (10^− 5^ < *p* < 5 × 10^− 8^) and SNPs at the genome wide level of statistical significance (*p* < 5 × 10^− 8^) may have a similar proportions of risk associated SNPs.

**Conclusions:**

The results of this study indicate that the SNP’s eQTL status, as well as eQTL density in the adjacent region are positively associated with the level of statistical significance of the SNP in GWAS.

## Background

Genome wide association studies (GWASs) have identified thousands of single nucleotide polymorphisms (SNPs) associated with human diseases [1]. Nevertheless, many disease-associated SNPs remain to be identified, which is obvious from the fact that larger GWASs targeting the same phenotype as the original and smaller GWAS regularly identify additional SNPs [2–4]. Those additional SNPs are usually SNPs from the gray zone of the original GWAS: gray zone SNPs are SNPs that are nominally significant (*p* < 0.05) but do not reach the genome-wide level of statistical significance (*p* < 5 × 10^− 8^) [5, 6]. It is, therefore, important to have a tool for prioritizing gray zone SNPs based on intrinsic SNP characteristics. A number of SNP characteristics including variation in allele frequencies among populations [7], type of the linked gene(s) [8], or combination of different SNP characteristics [9] were proposed for SNP prioritization. No GWAS of diseases have reached a sample size at which an exhaustive evaluation of all the possible genes or SNPs associated with disease can be anticipated to uncover all of the variability influence disease development and only studies of selected phenotypes like height and smoking behavior have amassed sample sizes that can provide comprehensive analyses of genetic architecture.

Conducting large enough GWAS studies to identify all the disease-associated SNPs, especially those with small effect sizes may not be feasible. Combining small effect SNPs in polygenic score is a useful approach for risk prediction [10, 11]. Interestingly, polygenic risk modeling performs better when the threshold for inclusion of a SNP is lower than genome-wide significance [12]. The accuracy of polygenic risk modeling will be reduced when a proportion of the variants being included are not associated with disease [13].

It is known that SNPs located in regulatory regions, e.g. transcription factor (TF) binding sites, are often eQTLs, as they modulate gene expression [14, 15]. A number of studies report an association between eQTLs and GWAS detected SNPs [16–19]. A systematic review of SNP eQTL status in the context of GWAS statistical significance has not been conducted so far. The goal of this study was to use summary statistics from recently published large breast and lung cancer GWASs to analyze the associations between the level of statistical significance of the SNP and its eQTL status. We also studied the association between the level of statistical significance of the SNP and eQTL density in adjacent region of the SNP.

## Methods

### Retrieving data on the SNP’ eQTL status

We used eQTLs reported by the Genotype-Tissue Expression (GTEx) project [20]. eQTL data were downloaded from the GTEx website (accessed October 12, 2018). Only cis eQTLs were used in the current analyses. To ensure robustness of the analysis, only eQTLs whose association with gene expression level remained significant after adjustment for multiple testing were used in the analysis. A total of 297,470 unique eQTLs detected in at least one out of 48 tissues analyzed by GTEx were used in the analysis. Additional file 1: Table S1 shows distribution of GTEx tested SNPs by tissue. More than 80% of eQTLs are tissue specific. Adjustment for multiple testing was done for each tissue separately based on the number of statistical tests. We used Bonferroni correction with significance level after adjustment 0.05.

All 48 tissue types available through GTEx were used in the analysis. Even though some tissues are certainly related, for example there are 13 tissues from different brain areas and 3 artery-derived tissues: artery aorta, artery coronary, and artery tibial all were analyzed separately as it was done by GTEx.

### GWAS SNPs

We have used summary statistics from breast and lung cancer OncoArray GWASs [21, 22]. Those two studies were selected because for both of them complete summary statistics were readily available. The breast GWAS summary statistics were downloaded from the Genome-Wide Repository of Associations Between SNPs and Phenotypes (GRASP) database [23]. Summary statistics for lung cancer GWAS is available for downloading from dbGaP: accession number phs001273.v1.p1. The sample size for the breast cancer GWAS was 122,977 cases and 105,974 controls. Lung OncoArray study included 29,266 cases and 56,450 controls. The studies analyzed over 500 K SNPs directly genotyped by the OncoArray [24]. Directly genotyped SNPs include candidate SNPs for breast, colorectal, lung, ovarian and prostate cancers. The platform also includes ~ 276 K backbone tag SNPs selected by OncoArray consortium to ensure reliable imputation of additional SNP [24]. Backbone SNPs are used as tag SNPs for imputation. Backbone SNPs are uniformly distributed across genome and generally show less linkage compared to all (directly genotyped plus imputed) OncoArray SNPs. We also used summary statistics from the scleroderma GWAS [25]. Scleroderma, or systemic sclerosis, is an autoimmune disease characterized by fibrosis of the skin and internal organs.

There was a substantial overlap between GTEx tested SNPs (Illumina OMNI 2.5 M SNP Array) and SNPs genotyped or imputed by breast and lung GWASs: 91% eQTLs were tested in breast and 92% in lung cancer GWAS. Only SNPs genotyped/imputed by both GTEx and the GWASs were used in the analysis. As a measure of statistical significance we have used –log_10_p where p is *p*-value.

### Statistical analysis

Non-parametric Mann-Whitney U test was used to compare -log_10_p(s) between eQTL and non-eQTL GWAS SNPs. To illustrate the relationship between eQTLs and the level of statistical significance (−log_10_p) in stratified analyses (Figs. 2 and 4) we used means and standard error of mean (SE). For correlation analyses we have used Spearman rank order correlation tests. All statistical tests were implemented in Statistica (TIBCO Software Inc., 2017).

## Results

### SNP’s eQTL status and the level of statistical significance in GWAS

Nominally significant breast cancer GWAS SNPs were used in this analysis. Tables 1 and 2 as well as Additional file 1: Tables S1-S3 show mean -log_10_p for the SNPs that are reported as eQTL versus SNPs that are not eQTLs in a given tissue. eQTL SNPs had higher -log_10_p regardless of the tissue specificity of the eQTL. We expected that breast tissue eQTLs will show the strongest -log_10_p inflation, based on the larger sample size of the original study to identify GWAS SNPs. We found, however, that breast eQTLs showed an average level of statistical significance compared to eQTLs for other tissue types.

**Table 1:** Mean -log_10_p for non-eQTL and eQTL breast cancer OncoArray SNPs. The eQTLs are stratified by tissue types. Only gray zone SNPs (0.05 > *p* > 5 × 10^− 8^) were used in this analysis

eQTL tissue type	non-eQTL SNP		eQTL SNP		^a^MW(Z)	*P*
	-log10p^b^	N	-log10p^b^	N		
Esophagus Mucosa	2.01/1.83	1,044,642	2.27/2.04	1556	11.99	2.56E-32
Thyroid	2.01/1.83	1,044,339	2.24/2.01	1859	11.55	4.40E-30
Nerve Tibial	2.01/1.83	1,044,334	2.22/2	1864	10.64	1.02E-25
Pancreas	2.01/1.83	1,045,104	2.28/2.05	1094	10.42	1.03E-24
Artery Aorta	2.01/1.83	1,044,905	2.25/2.02	1293	10.34	2.45E-24
Skin Not Sun Exposed Suprapubic	2.01/1.83	1,044,670	2.23/2.01	1528	10.16	1.56E-23
Lung	2.01/1.83	1,044,615	2.22/2	1583	10.1	2.80E-23
Stomach	2.01/1.83	1,045,249	2.28/2.05	949	9.75	8.90E-22
Adipose Subcutaneous	2.01/1.83	1,044,510	2.21/1.99	1688	9.75	9.52E-22
Muscle Skeletal	2.01/1.83	1,044,683	2.22/2	1515	9.74	9.64E-22
Esophagus Muscularis	2.01/1.83	1,044,642	2.22/2	1556	9.6	4.00E-21
Testis	2.01/1.83	1,044,391	2.2/1.98	1807	9.39	2.77E-20
Esophagus Gastroesophageal Junction	2.01/1.83	1,045,177	2.26/2.03	1021	9.36	3.63E-20
Colon Sigmoid	2.01/1.83	1,045,157	2.25/2.02	1041	9.35	4.16E-20
Skin Sun Exposed Lower leg	2.01/1.83	1,044,398	2.19/1.97	1800	9.15	2.53E-19
Adipose Visceral Omentum	2.01/1.83	1,044,921	2.23/2.01	1277	9.14	2.89E-19
Heart Left Ventricle	2.01/1.83	1,045,132	2.24/2.01	1066	8.94	1.70E-18
Cells Transformed fibroblasts	2.01/1.83	1,044,639	2.2/1.98	1559	8.88	2.98E-18
Artery Coronary	2.01/1.83	1,045,557	2.3/2.06	641	8.78	7.49E-18
Artery Tibial	2.01/1.83	1,044,545	2.19/1.97	1653	8.58	3.99E-17
Brain Cerebellum	2.01/1.83	1,045,002	2.21/1.99	1196	8.38	2.29E-16
Breast Mammary Tissue	2.01/1.83	1,045,173	2.23/2.01	1025	8.35	2.81E-16
Pituitary	2.01/1.83	1,045,266	2.23/2.01	932	8.13	1.82E-15
Adrenal Gland	2.01/1.83	1,045,270	2.23/2.01	928	7.99	5.29E-15
Heart Atrial Appendage	2.01/1.83	1,045,051	2.2/1.98	1147	7.75	3.56E-14
Colon Transverse	2.01/1.83	1,045,071	2.21/1.99	1127	7.75	3.71E-14
Cells EBV-transformed lymphocytes	2.01/1.83	1,045,617	2.28/2.05	581	7.74	3.81E-14
Whole Blood	2.01/1.83	1,045,014	2.2/1.98	1184	7.71	5.10E-14
Spleen	2.01/1.83	1,045,243	2.21/1.99	955	7.29	1.17E-12
Liver	2.01/1.83	1,045,581	2.25/2.02	617	7	9.12E-12
Brain Hypothalamus	2.01/1.83	1,045,693	2.27/2.04	505	6.9	1.79E-11
Prostate	2.01/1.83	1,045,571	2.23/2.01	627	6.65	1.01E-10
Brain Cerebellar Hemisphere	2.01/1.83	1,045,174	2.19/1.97	1024	6.64	1.08E-10
Brain Putamen basal ganglia	2.01/1.83	1,045,561	2.23/2.01	637	6.6	1.36E-10
Brain Caudate basal ganglia	2.01/1.83	1,045,364	2.19/1.97	834	6.24	1.44E-09
Brain Amygdala	2.01/1.83	1,045,805	2.27/2.04	393	6.21	1.72E-09
Vagina	2.01/1.83	1,045,803	2.25/2.02	395	5.7	3.61E-08
Brain Substantia nigra	2.01/1.83	1,045,894	2.28/2.05	304	5.54	8.85E-08
Ovary	2.01/1.83	1,045,627	2.2/1.98	571	5.42	1.66E-07
Brain Frontal Cortex BA9	2.01/1.83	1,045,459	2.18/1.97	739	5.37	2.13E-07
Minor Salivary Gland	2.01/1.83	1,045,836	2.25/2.02	362	5.36	2.25E-07
Brain Anterior cingulate cortex BA24	2.01/1.83	1,045,520	2.18/1.97	678	5.21	5.21E-07
Brain Nucleus accumbens basal ganglia	2.01/1.83	1,045,408	2.17/1.96	790	5.2	5.41E-07
Brain Spinal cord cervical c-1	2.01/1.83	1,045,737	2.2/1.98	461	4.77	4.66E-06
Small Intestine Terminal Ileum	2.01/1.83	1,045,503	2.16/1.95	695	4.66	7.51E-06
Brain Cortex	2.01/1.83	1,045,253	2.13/1.93	945	4.31	3.74E-05
Uterus	2.01/1.83	1,045,780	2.18/1.97	418	4.09	9.19E-05
Brain Hippocampus	2.01/1.83	1,045,658	2.16/1.95	540	4.04	1.12E-04

^a^MW(Z) is a Z statistics from Mann-Whitney test for comparing two samples

^b^mean/median

**Table 2:** Mean -log_10_p for not eQTL and eQTL lung cancer OncoArray SNPs. The eQTLs are stratified by tissue types

eQTL tissue type	non-eQTL SNP		eQTL SNP		^a^MW(Z)	*P*
	-log10p^b^	N	-log10p^b^	N		
Uterus	1.88/1.69	798,995	2.26/2.01	269	8.62	2.90E-17
Adipose Subcutaneous	1.88/1.69	798,297	2.04/1.83	967	7.98	5.90E-15
Vagina	1.88/1.69	798,997	2.23/1.99	267	7.95	7.60E-15
Spleen	1.88/1.69	798,688	2.13/1.9	576	7.92	9.90E-15
Prostate	1.88/1.69	798,847	2.16/1.93	417	7.78	2.80E-14
Ovary	1.88/1.69	798,916	2.18/1.95	348	7.72	4.40E-14
Cells EBV-transformed lymphocytes	1.88/1.69	798,893	2.18/1.95	371	7.69	5.90E-14
Pituitary	1.88/1.69	798,733	2.12/1.89	531	7.54	1.70E-13
Artery Coronary	1.88/1.69	798,862	2.12/1.89	402	7.54	1.80E-13
Small Intestine Terminal Ileum	1.88/1.69	798,827	2.13/1.9	437	7.41	4.60E-13
Adipose Visceral Omentum	1.88/1.69	798,503	2.07/1.85	761	7.34	8.00E-13
Brain Substantia nigra	1.88/1.69	799,078	2.26/2.01	186	7.28	1.20E-12
Whole Blood	1.88/1.69	798,578	2.09/1.87	686	7.15	3.20E-12
Brain Spinal cord cervical c-1	1.88/1.69	798,979	2.2/1.96	285	7.09	4.90E-12
Stomach	1.88/1.69	798,724	2.11/1.89	540	7.06	5.80E-12
Breast Mammary Tissue	1.88/1.69	798,703	2.1/1.88	561	7.03	7.60E-12
Brain Putamen basal ganglia	1.88/1.69	798,889	2.14/1.91	375	6.99	9.50E-12
Brain Cerebellum	1.88/1.69	798,524	2.05/1.83	740	6.99	9.80E-12
Brain Nucleus accumbens basal ganglia	1.88/1.69	798,766	2.12/1.89	498	6.96	1.20E-11
Brain Cortex	1.88/1.69	798,735	2.11/1.89	529	6.9	1.80E-11
Esophagus Gastroesophageal Junction	1.88/1.69	798,628	2.09/1.87	636	6.83	3.00E-11
Artery Tibial	1.88/1.69	798,325	2.02/1.81	939	6.78	4.20E-11
Colon Transverse	1.88/1.69	798,567	2.08/1.86	697	6.71	6.70E-11
Pancreas	1.88/1.69	798,628	2.09/1.87	636	6.7	7.30E-11
Skin Not Sun Exposed Suprapubic	1.88/1.69	798,372	2.05/1.83	892	6.66	9.30E-11
Heart Atrial Appendage	1.88/1.69	798,574	2.08/1.86	690	6.6	1.30E-10
Adrenal Gland	1.88/1.69	798,712	2.06/1.84	552	6.59	1.50E-10
Brain Cerebellar Hemisphere	1.88/1.69	798,607	2.04/1.83	657	6.51	2.50E-10
Liver	1.88/1.69	798,893	2.11/1.89	371	6.47	3.20E-10
Brain Frontal Cortex BA9	1.88/1.69	798,788	2.1/1.88	476	6.31	9.00E-10
Skin Sun Exposed Lower leg	1.88/1.69	798,261	2.04/1.83	1003	6.27	1.20E-09
Brain Hippocampus	1.88/1.69	798,936	2.14/1.91	328	6.12	3.00E-09
Heart Left Ventricle	1.88/1.69	798,640	2.07/1.85	624	6.08	3.70E-09
Thyroid	1.88/1.69	798,130	2.02/1.81	1134	5.92	9.60E-09
Lung	1.88/1.69	798,354	2.02/1.81	910	5.88	1.20E-08
Nerve Tibial	1.88/1.69	798,217	2.02/1.81	1047	5.85	1.50E-08
Esophagus Mucosa	1.88/1.69	798,316	2.03/1.82	948	5.85	1.50E-08
Minor Salivary Gland	1.88/1.69	799,032	2.14/1.91	232	5.84	1.60E-08
Artery Aorta	1.88/1.69	798,498	2.01/1.8	766	5.69	3.70E-08
Brain Caudate basal ganglia	1.88/1.69	798,771	2.04/1.83	493	5.53	9.10E-08
Colon Sigmoid	1.88/1.69	798,658	2.05/1.83	606	5.45	1.40E-07
Testis	1.88/1.69	798,195	2.01/1.8	1069	5.44	1.50E-07
Brain Hypothalamus	1.88/1.69	798,938	2.12/1.89	326	5.4	1.80E-07
Muscle Skeletal	1.88/1.69	798,409	2.02/1.81	855	5.22	4.90E-07
Cells Transformed fibroblasts	1.88/1.69	798,375	2.02/1.81	889	5.06	1.10E-06
Brain Anterior cingulate cortex BA24	1.88/1.69	798,880	2.02/1.81	384	3.91	1.90E-04
Brain Amygdala	1.88/1.69	799,022	1.98/1.78	242	2.44	2.00E-02

^a^MW(Z) is a Z statistics from Mann-Whitney test for comparing two samples

^b^mean/median

Similar to breast cancer, in lung cancer GWAS we also found that the SNPs reported as eQTLs tended to be more significant regardless of the tissue specificity of the eQTL (Table 2). Lung tissue specific eQTLs were associated with an average (typical) inflation of -log_10_p compared to eQTLs specific for other tissue types.

### SNPs showing eQTL activity in multiple tissues (pan-tissue eQTLs) exhibit a higher -log_10_p inflation

eQTLs can be roughly divided into tissue specific (those reported as an eQTL in a single tissue type) and pan-tissue eQTLs – those showing eQTL activity across multiple tissues. Additional file 1: Table S1 shows the distribution of eQTL SNPs by the number of tissues where they are reported. Over 80% of eQTLs are tissue-specific while only a few SNPs show eQTL activity across all 48 tissues.

**Fig. 1:**
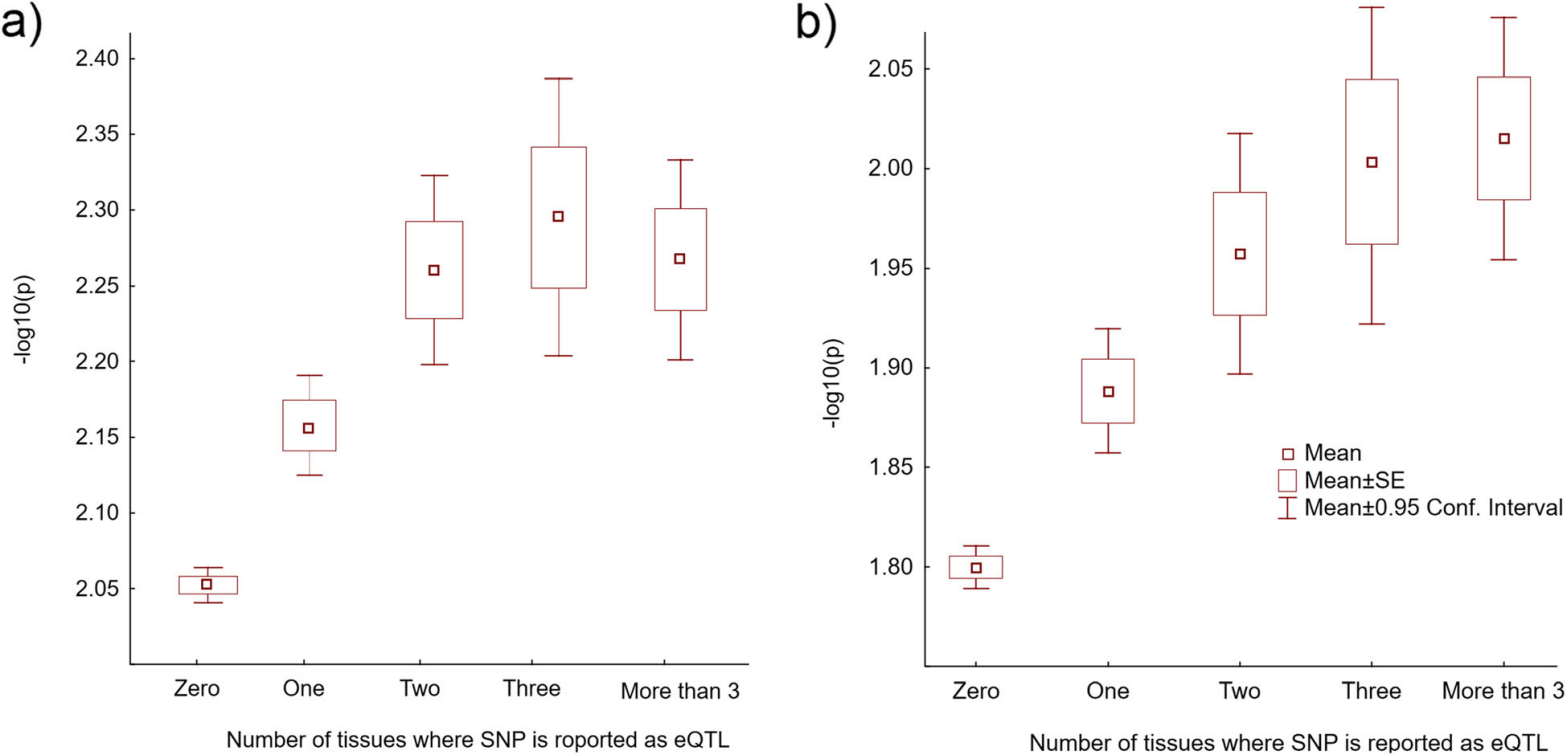
Mean -log_10_p in SNPs stratified by the number of tissues where it is reported as an eQTL. **a** Breast cancer GWAS; **b** Lung cancer GWAS

GWAS SNPs were subdivided into 5 categories based on the number of tissues where a SNP is reported as eQTL: “0”, “1”, “2”, “3” and “> 3” and mean -log_10_ps were computed in each category (Fig. 1). Figure 1a shows the result of the analyses of breast cancer GWAS SNPs and Fig. 1b - lung cancer GWAS SNPs. In both studies pan-tissue eQTLs show a higher inflation of -log_10_p compared to tissue specific eQTLs.

### Statistical significance of a SNP in GWA studies is positively associated with the number of eQTLs in its adjacent region

**Fig. 2:**
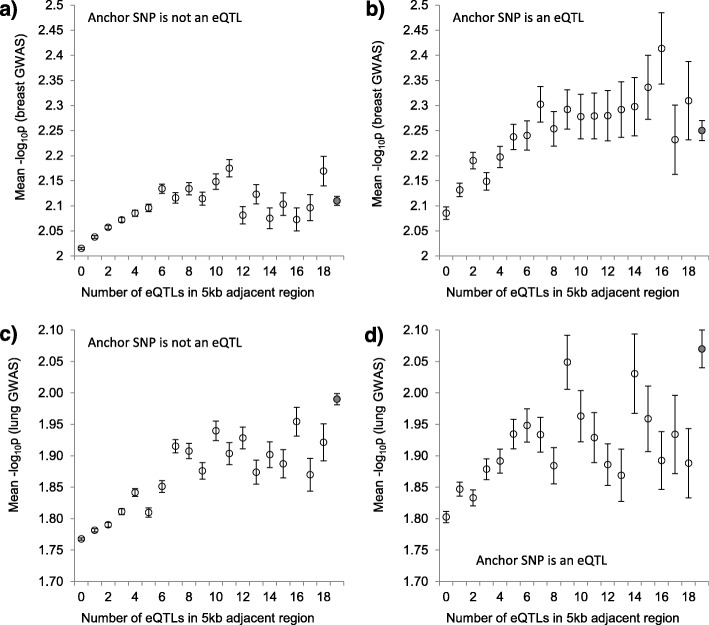
The relationship between the number of eQTLs in ±5 kb adjacent region of the anchor SNP and the level of statistical significance. **a** Breast cancer gray zone SNPs that are not eQTLs. **b** Gray zone SNPs from breast cancer GWAS that are eQTLs. **c** Gray zone SNPs from lung cancer GWAS that are not eQTLs. **d**. Gray zone SNPs from lung cancer GWAS that are eQTLs. Vertical bars show standard error (SE) of the mean

We tested if the density of eQTLs in the adjacent ±5 kb region is associated with the level of statistical significance of the SNP in GWASs. The size of the adjacent region was selected because it is a typical size of haplotype blocks in the human genome [26]. SNPs were categorized by the number of eQTLs in the adjacent region and mean -log_10_p were estimated for each category (Fig. 2). We found that -log_10_p(s) for breast cancer (upper panel) SNPs were positively associated with the number of eQTLs in adjacent regions. There was a linear association in 0–6 eQTLs interval and after that the curve plateaued. For SNPs that themselves are not eQTLs (a) and eQTLs (b) the associations were similar. The results for lung cancer GWAS (c, d) were similar to the breast cancer GWAS results.

### SNPs with a higher density of eQTLs in adjacent region are more likely to be located in regulatory regions

**Fig. 3:**
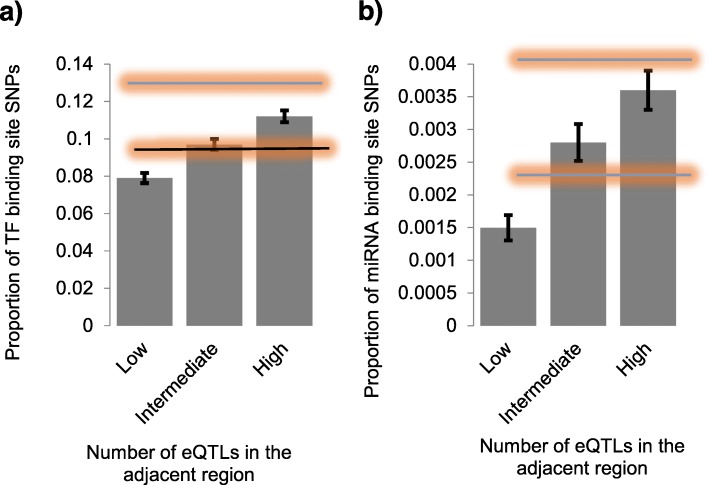
The proportion of transcription factor (**a**) and miRNA (**b**) binding sites in SNPs categorized by low, intermediate and high density of eQTLs in ±5 kb adjacent region. Vertical bars represent standard errors of mean. Horizontal black lines represent the proportion of non- eQTL SNPs co-localizing with TF binding sites (**a**) and miRNA binding sites (**b**). Horizontal blue lines represent the proportion of eQTL SNPs co-localizing with TF and miRNA binding sites. Orange areas represent SE of means for the analysis. Black vertical bars show standard error (SE) of the mean

High eQTL density may be indicative of high density of regulatory elements in the region. Based on the density of eQTLs in the adjacent region we subdivided GWAS SNPs into three categories: low density (no eQTLs detected in ±5 kb region), intermediate density (1–7 eQTLs); and high density – eight or more eQTLs in the adjacent region of the anchor SNP. The cut points for these categories were chosen to ensure similar sizes of the groups. Encyclopedia of DNA Elements (ENCODE) data [27] were used to identify transcription factor (TF) and miRNA binding sites [28]. Figure 3 shows the proportions of SNPs co-localizing with TF (3a) and miRNA binding sites (3b) among GWAS SNPs with low, intermediate and high density of eQTLs in adjacent regions. We found that SNPs with high density of eQTLs in adjacent regions are more likely to be located in regulatory regions.

The comparison of all eQTL SNPs (blue lines on Fig. 3) versus all non eQTL SNPs (black lines on Fig. 3) shows that eQTL SNPs are more likely to co-localize with TF binding sites (Fig. 3a) as well as miRNA binding sites (Fig. 3b).

### Genome chopping

The detected positive association between eQTL density in adjacent regions of the SNPs and its level of statistical significance in GWAS can potentially be biased because of a non-uniform distribution of SNPs along chromosomes. If the SNP density is higher in a region of GWAS peaks, the 5 kb regions of the many SNPs located in the peak will overlap and, as a result, be overrepresented in the analysis. To assess the association between eQTL density and the level of statistical significance in non-overlapping regions we divided the human genome into consecutive (non-overlapping) 5 kb fragments starting from the first nucleotide of each chromosome. The total number of fragments was 558,455. About 70% of fragments (total 391,824) do not contain eQTLs. The highest number of eQTLs was detected in a fragment on chromosome 5, position 570,305,043–70,310,042 – 117 eQTLs. Additional file 1: Figure S1a shows the distribution of the fragments by the number of eQTLs in them. Additional file 1: Figure S1b shows the distribution of fragments by the number of genotyped lung cancer GWAS SNPs in them. The mean and median numbers of SNPs per fragment are correspondingly 35.1 and 33. Similar results were obtained for breast cancer GWAS SNPs (Additional file 1: Figure S1c).

We observed a significant positive association between the number of eQTLs in non-overlapping fragments and the mean -log_10_p for the breast cancer GWAS SNPs from the corresponding fragments (Spearman Rank Order correlation *R* = 0.05, df = 558,455, *p* = 7.5 × 10^− 28^). The correlation remained significant after the exclusion of all fragments with at least one genome wide significant SNP (Spearman Rank Order correlation *R* = 0.05, df = 554,672, *p* = 3.4 × 10^− 26^). A similar association was observed for lung GWAS SNPs: *R* = 0.03, df = 558,455, *p* = 3.6 × 10^− 18^. The correlation remained significant after the exclusion of all fragments with at least one genome wide significant SNP (Spearman Rank Order correlation *R* = 0.03, df = 557,675, *p* = 3.9 × 10^− 16^).

### Proportions of eQTLs in groups of SNPs categorized by the level of statistical significance in GWAS

The results of several studies suggest that eQTLs are more likely to be causal, risk-associated SNPs compared to non-eQTL SNPs [29–32]. If it is true, the proportion of eQTLs among more significant SNPs is expected to be higher. We subdivided GWAS SNPs into 4 categories based on the level of their statistical significance and estimated proportions of eQTLs in them: *white noise* – SNPs that do not reach the level of nominal significance *p* > 0.05; *light gray* SNPs – SNPs in the lower part of the gray zone those that are nominally significant but do not reach genome wide level of statistical significance 0.05 > *p* > 5 × 10^− 5^; *dark gray* SNPs – SNPs in the upper part of the gray zone: 5 × 10^− 5^ > *p* > 5 × 10^− 8^, and genome wide *(GW) significant* SNPs – those with *p* < 5 × 10^− 8^. We estimated the proportions of eQTLs in each category.

**Fig. 4:**
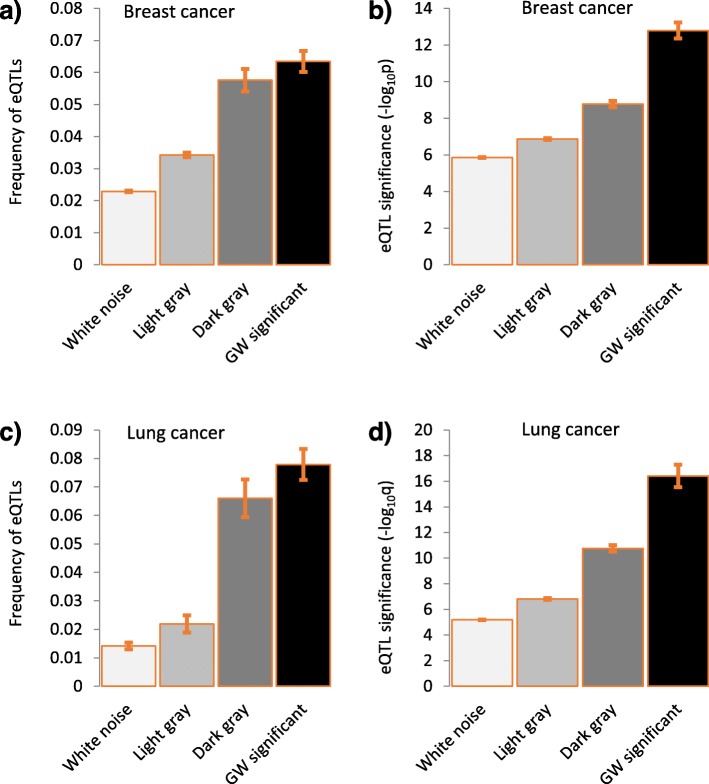
The proportions (**a** and **c**) and significance (**b** and **d**) of eQTLs among SNPs from breast (upper panel) and lung (lower panel) cancer GWASs categorized by the level of statistical significance. Vertical bars show standard error (SE) of the mean

The proportions of eQTLs were higher among more significant SNPs (Fig. 4a and c). We noted that eQTLs not only became more frequent as GWAS significance level went up, but they as well became more significant themselves: -log_10_q (where q is the *p* value for association between the number of variant alleles and gene expression adjusted for multiple testing) significantly increases from white noise to GW significant SNPs (Fig. 4b and d).

**Fig. 5:**
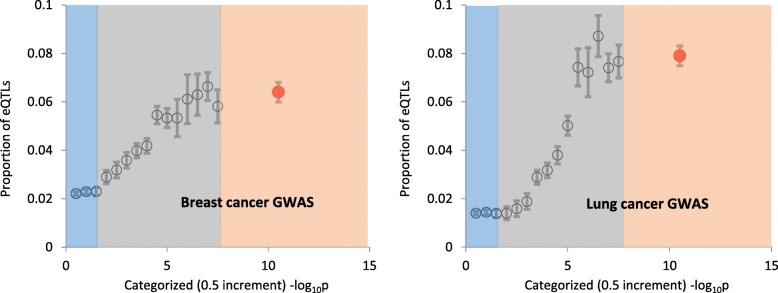
Proportions of eQTLs in breast (left panel) and lung (right panel) cancer GWAS SNPs categorized based on -log_10_p with 0.5 increments. Blue shade indicates non-significant SNPs (*p* > 0.05), gray shade - gray zone SNPs (0.05 > *p* > 5 × 10^− 8^), and orange - SNPs significant at the genome-wide level (*p* < 5 × 10^− 8^). Vertical bars show standard error (SE) of the mean

For a more granular analysis we categorized GWAS SNPs based on the level of statistical significance using 0.5 increments for -log_10_p (16 categories in total). Figure 5, left panel, shows results for breast cancer, and right – for lung cancer GWAS. For nonsignificant SNPs (blue-shaded areas - those with -log_10_p between 0 and 1.3), the proportion of eQTLs was low and flat across all categories, with the average proportion of eQTLs 1.40 ± 0.01% in lung cancer and ~ 2.11 ± 0.01 in breast cancer. For breast cancer GWAS gray zone SNPs (those with -log_10_p between 1.3 and 7.3), the average percentage of eQTLs was 2.14 ± 0.01%. For gray zone SNPs we observed a significant positive association between the proportion of eQTLs and -log_10_p (Spearman rank order correlation *R* = 0.95, *N* = 12, *p* = 2.1 × 10^− 6^). Since the number of SNPs with genome wide level of statistical significance is relatively small, we combined them together. For lung cancer GWAS, the association between the level of GWAS significance and the proportion of eQTLs was similar to that in the breast cancer GWAS.

### Analysis of backbone SNPs

The results generated by analyses of backbone SNPs were similar to those generated by the analyses of all OncoArray SNPs. Regardless of their tissue specificity backbone eQTL SNPs tended to be more significant than non-eQTL SNPs in both breast and lung GWASs (Additional file 1: Tables S2 and S3). Densities of eQTLs in the 5 kb adjacent region were positively associated with -log_10_p in both breast and lung backbone SNPs (Additional file 1: Figure S2).

### Scleroderma GWAS

We analyzed summary statistics from scleroderma GWAS to check if findings from cancer GWASs hold for noncancerous disease. The results of the analysis of association between a SNP’s eQTL status and the level of statistical significance in scleroderma GWAS were similar to the results for breast and lung cancer GWASs. SNPs reported as eQTLs tended to be more significant in scleroderma GWAS than non-eQTL SNPs regardless of the tissue specificity (Additional file 1: Table S4). Similar to the analyses of breast and lung GWASs we found that eQTL density in the adjacent ±5 kb region was positively associated with the level of statistical significance (Additional file 1: Figure S3).

## Discussion

We found that GWAS eQTL SNPs tended to be more significant compared to non-eQTL SNPs. Tissue-specific eQTLs (breast and lung eQTLs in this analysis) did not show a higher level of inflation in significance level compared to other tissues. The likely reason for the lack of tissue specificity may be that eQTLs often show multiple-tissue effects. Almost 20% of eQTLs have more than one target tissue. An overlap across different tissue types is stronger when less stringent criteria to define eQTLs are used [33]. When a SNP acts as a eQTL in multiple tissue types, the direction of the effect is the same in more than 97% cases [33]. Based on this observation one can suggest that eQTLs with a significant effect on gene expression in one tissue type often have a similar effect in other tissue types. eQTLs with pan-tissue effects are not currently very common because they may not have been all identified due to the small sample size of GTEx [20].

We found that the level of statistical significance of a SNP in GWAS is positively associated with the eQTL density in its adjacent region regardless of its eQTL status. We think that the reason for these associations can be that some SNPs that are not reported as eQTLs are, in fact, eQTLs (false negatives). This suggestion is further supported by the observation that non-eQTL SNPs located in eQTL-rich regions often co-localize with regulatory regions.

We also noted that the proportion of eQTLs increases with the increasing level of statistical significance in GWAS and reaches plateau at the level of ~ 10^− 5^–10^− 6^. The simplest explanation to this can be that eQTLs have a higher probability to be causal, risk-associated SNPs and as a result categories with a high level of statistical significance have a higher proportion of eQTL SNPs. Proportions of eQTLs in the group may reflect the proportion of true positives. This analysis found that the proportion of eQTLs plateaued at the level of statistical significance about 10^− 5^, suggesting that the proportions of causal SNPs may be similar among dark gray SNPs and SNPs at the genome-wide level of statistical significance.

It is likely that the associations found between eQTL status/density and the level of statistical significance in cancer GWASs also hold for other phenotypes. This is supported by analysis of summary statistics for scleroderma GWAS. F.

The effect size of the association between eQTLs and the level of statistical significance was relatively small. This suggests that although the eQTL status of the SNP as well as eQTL density in the surrounding region can be useful in SNP prioritizing it would be better to use them in combination with other SNP characteristics associated with functionality, e.g. the level of evolutionary conservation of the site [34]. The limitation of our analysis is that -log_10_p is study specific (GWASs with a larger sample size are likely to have a larger for -log_10_ps) which makes it difficult to generalize exact shapes of SNP/eQTL relationships.

The major findings of this study are:

1. eQTL SNPs are more significant in GWASs regardless of their tissue specificity;
2. Pan-tissue eQTLs are associated with a higher inflation of -log_10_p compared to tissue specific eQTLs;
3. SNPs located in regions of high eQTL density are more significant in GWAS regardless of their own eQTL status;
4. The probability of a SNP to be an eQTL is positively associated with the level of statistical significance in a GWAS. The association curve plateaued after -log_10_p~ 5 suggesting that SNPs from the dark gray zone (10^− 5^ > *p* > 5 × 10^− 8^) and SNPs at the genome wide level of statistical significance have a similar proportion of causal SNPs.

## Conclusions

Our results suggest that a substantial subset of SNPs in the dark grey zone are eQTLs and therefore likely to be causally associated with disease development. Causal risk-associated SNPs from dark gray zone may not be detected by GWAS because of their smaller effect size and the limited sample sizes available from most GWAS studies. Nevertheless, SNPs that are associated with an increased risk for disease development should be included as a part of the polygenic risk score modeling process. Results that we have obtained suggest prioritizing SNPs for polygenic risk score modeling that are strongly or moderately associated with disease risk and act as eQTLs, particularly in multiple tissues.

## Supplementary information

**Additional file 1: Table S1.** Distribution of eQTL SNPs by the number of tissues where they are reported as eQTLs. eQTL SNPs with “Number of tissues” equal to one are tissue specific; others are pan-tissue. **Table S2.** Mean -log_10_p for non-eQTL and eQTL backbone breast cancer OncoArray SNPs. The eQTLs are stratified by tissue types. **Table S3.** Mean -log_10_p for non-eQTL and eQTL backbone lung cancer OncoArray SNPs. The eQTLs are stratified by tissue types. **Table S4.** Mean -log_10_p for non-eQTL and eQTL scleroderma GWAS SNPs. The eQTLs are stratified by tissue types. **Figure S1.** a) The distribution of non-overlapping 5 kb fragments by the number of eQTLs. b) The distribution of 5 kb non-overlapping chromosomal fragments by the number of lung GWAS SNPs. c) Distribution of 5 kb non-overlapping chromosomal fragments by the number of breast cancer GWAS SNPs. **Figure S2.** The relationship between the number of eQTLs in the ±5 kb adjacent region and the level of statistical significance of the backbone SNP. **a.** Breast cancer GWAS SNPs. **b.** Lung cancer GWAS SNPs. Shaded circle indicate SNPs with > 18 eQTLs in ±5 kb adjacent region. Vertical bars show standard error (SE) of the mean. **Figure S3.** The relationship between the number of eQTLs in ±5 kb adjacent region and the level of statistical significance of scleroderma SNP. Shaded circle indicate SNPs with > 18 eQTLs in ±5 kb adjacent region. Vertical bars show standard error (SE) of the mean.

## Data Availability

All relevant data are contained within the paper and its Additional files. eQTL data were downloaded from GTEx website: https://gtexportal.org/home/ Breast OncoArray GWAS summary statistics data were downloaded from Genome-Wide Repository of Associations Between SNPs and Phenotypes (GRASP) database. https://grasp.nhlbi.nih.gov/Overview.aspx Summary statistics for scleroderma GWAS available from MM upon request. Summary statistics for lung cancer GWAS available from the database of Genotypes and Phenotypes (dbGaP): accession number phs001273.v1.p1.

## Abbreviations

ENCODE: Encyclopedia of DNA elements
eQTL: Expression quantitative trait loci
GRASP: Genome-Wide Repository of Associations Between SNPs and Phenotypes
GTEx: Genotype-Tissue Expression project
GWAs: Genome wide association studies
SNP: Single nucleotide polymorphism
