# Supplementary material for: SNP eQTL status and eQTL density in the adjacent region of the SNP are associated with its statistical significance in GWA studies
Source: BMC Genet. 2019 Nov 12;20:85. doi: 10.1186/s12863-019-0786-0 (PMC6852916; doi:10.1186/s12863-019-0786-0)

**Additional file 1**

**Table S1.** Distribution of eQTL SNPs by the number of tissues where they are reported as eQTLs. eQTL SNPs with “Number of tissues” equal to one are tissue specific; others are pan-tissue.

| Number of tissues where a SNP reported as eQTL | Number of eQTLs | % of all eQTLs |
| --- | --- | --- |
| 1 | 246335 | 82.810 |
| 2 | 29853 | 10.036 |
| 3 | 9128 | 3.069 |
| 4 | 4087 | 1.374 |
| 5 | 2276 | 0.765 |
| 6 | 1363 | 0.458 |
| 7 | 939 | 0.316 |
| 8 | 595 | 0.200 |
| 9 | 495 | 0.166 |
| 10 | 385 | 0.129 |
| 11 | 288 | 0.097 |
| 12 | 235 | 0.079 |
| 13 | 188 | 0.063 |
| 14 | 161 | 0.054 |
| 15 | 150 | 0.050 |
| 16 | 115 | 0.039 |
| 17 | 111 | 0.037 |
| 18 | 79 | 0.027 |
| 19 | 68 | 0.023 |
| 20 | 68 | 0.023 |
| 21 | 58 | 0.019 |
| 22 | 47 | 0.016 |
| 23 | 39 | 0.013 |
| 24 | 37 | 0.012 |
| 25 | 36 | 0.012 |
| 26 | 37 | 0.012 |
| 27 | 30 | 0.010 |
| 28 | 36 | 0.012 |
| 29 | 21 | 0.007 |
| 30 | 23 | 0.008 |
| 31 | 20 | 0.007 |
| 32 | 17 | 0.006 |
| 33 | 15 | 0.005 |
| 34 | 4 | 0.001 |
| 35 | 13 | 0.004 |
| 36 | 16 | 0.005 |
| 37 | 13 | 0.004 |
| 38 | 14 | 0.005 |
| 39 | 11 | 0.004 |
| 40 | 14 | 0.005 |
| 41 | 7 | 0.002 |
| 42 | 6 | 0.002 |
| 43 | 7 | 0.002 |
| 44 | 5 | 0.002 |
| 45 | 9 | 0.003 |
| 46 | 8 | 0.003 |
| 47 | 4 | 0.001 |
| 48 | 4 | 0.001 |

**Table S2.** Mean -log_10_p for non-eQTL and eQTL backbone breast cancer OncoArray SNPs. The eQTLs are stratified by tissue types.

| eQTL tissue type | non-eQTL SNP | | eQTL SNP | | *MW(Z) | p |
| --- | --- | --- | --- | --- | --- | --- |
|  | **-log10p | N | **-log10p | N |  |  |
| Adipose Subcutaneous | 0.63/0.38 | 131681 | 0.78/0.52 | 417 | 2.23 | 3.00E-02 |
| Adipose Visceral Omentum | 0.63/0.38 | 131767 | 0.61/0.36 | 331 | 0.3 | 7.60E-01 |
| Adrenal Gland | 0.63/0.38 | 131867 | 0.73/0.47 | 231 | 1.03 | 3.00E-01 |
| Artery Aorta | 0.63/0.38 | 131755 | 0.73/0.47 | 343 | 1.23 | 2.20E-01 |
| Artery Coronary | 0.63/0.38 | 131940 | 0.67/0.42 | 158 | 0.25 | 8.00E-01 |
| Artery Tibial | 0.63/0.38 | 131659 | 0.66/0.41 | 439 | 0.37 | 7.10E-01 |
| Brain Amygdala | 0.63/0.38 | 132025 | 0.82/0.55 | 73 | 1.18 | 2.30E-01 |
| Brain Anterior cingulate cortex BA24 | 0.63/0.38 | 131938 | 0.92/0.64 | 160 | 2.68 | 1.00E-02 |
| Brain Caudate basal ganglia | 0.63/0.38 | 131903 | 0.7/0.44 | 195 | 0.63 | 5.20E-01 |
| Brain Cerebellar Hemisphere | 0.63/0.38 | 131856 | 0.7/0.44 | 242 | 0.68 | 4.90E-01 |
| Brain Cerebellum | 0.63/0.38 | 131771 | 0.74/0.48 | 327 | 1.38 | 1.60E-01 |
| Brain Cortex | 0.63/0.38 | 131870 | 0.68/0.43 | 228 | 0.53 | 5.90E-01 |
| Brain Frontal Cortex BA9 | 0.63/0.38 | 131911 | 0.7/0.44 | 187 | 0.67 | 4.90E-01 |
| Brain Hippocampus | 0.63/0.38 | 131967 | 0.72/0.46 | 131 | 0.66 | 5.10E-01 |
| Brain Hypothalamus | 0.63/0.38 | 131991 | 0.67/0.42 | 107 | 0.27 | 7.80E-01 |
| Brain Nucleus accumbens basal ganglia | 0.63/0.38 | 131903 | 0.69/0.43 | 195 | 0.54 | 5.90E-01 |
| Brain Putamen basal ganglia | 0.63/0.38 | 131950 | 0.71/0.45 | 148 | 0.69 | 4.80E-01 |
| Brain Spinal cord cervical c-1 | 0.63/0.38 | 132003 | 0.7/0.44 | 95 | 0.43 | 6.60E-01 |
| Brain Substantia nigra | 0.63/0.38 | 132020 | 0.83/0.56 | 78 | 1.28 | 2.00E-01 |
| Breast Mammary Tissue | 0.63/0.38 | 131840 | 0.79/0.52 | 258 | 1.84 | 6.00E-02 |
| Cells EBV-transformed lymphocytes | 0.63/0.38 | 131960 | 0.75/0.49 | 138 | 0.95 | 3.40E-01 |
| Cells Transformed fibroblasts | 0.63/0.38 | 131708 | 0.76/0.5 | 390 | 1.89 | 6.00E-02 |
| Colon Sigmoid | 0.63/0.38 | 131828 | 0.68/0.43 | 270 | 0.46 | 6.30E-01 |
| Colon Transverse | 0.63/0.38 | 131807 | 0.64/0.39 | 291 | 0.07 | 9.40E-01 |
| Esophagus Gastroesophageal Junction | 0.63/0.38 | 131872 | 0.81/0.54 | 226 | 1.98 | 4.00E-02 |
| Esophagus Mucosa | 0.63/0.38 | 131661 | 0.7/0.44 | 437 | 0.89 | 3.70E-01 |
| Esophagus Muscularis | 0.63/0.38 | 131726 | 0.62/0.37 | 372 | 0.22 | 8.20E-01 |
| Heart Atrial Appendage | 0.63/0.38 | 131777 | 0.8/0.53 | 321 | 2.24 | 2.00E-02 |
| Heart Left Ventricle | 0.63/0.38 | 131829 | 0.85/0.58 | 269 | 2.51 | 1.00E-02 |
| Liver | 0.63/0.38 | 131922 | 0.81/0.54 | 176 | 1.78 | 7.00E-02 |
| Lung | 0.63/0.38 | 131688 | 0.72/0.46 | 410 | 1.29 | 1.90E-01 |
| Minor Salivary Gland | 0.63/0.38 | 132019 | 0.63/0.38 | 79 | 0.01 | 9.90E-01 |
| Muscle Skeletal | 0.63/0.38 | 131701 | 0.7/0.44 | 397 | 0.97 | 3.20E-01 |
| Nerve Tibial | 0.63/0.38 | 131638 | 0.63/0.38 | 460 | 0.14 | 8.90E-01 |
| Ovary | 0.63/0.38 | 131950 | 0.61/0.36 | 148 | 0.2 | 8.40E-01 |
| Pancreas | 0.63/0.38 | 131845 | 0.77/0.51 | 253 | 1.6 | 1.10E-01 |
| Pituitary | 0.63/0.38 | 131861 | 0.72/0.46 | 237 | 0.92 | 3.50E-01 |
| Prostate | 0.63/0.38 | 131936 | 0.65/0.4 | 162 | 0.13 | 8.90E-01 |
| Skin Not Sun Exposed Suprapubic | 0.63/0.38 | 131701 | 0.78/0.52 | 397 | 2.1 | 4.00E-02 |
| Skin Sun Exposed Lower leg | 0.63/0.38 | 131633 | 0.71/0.45 | 465 | 1.12 | 2.60E-01 |
| Small Intestine Terminal Ileum | 0.63/0.38 | 131941 | 0.71/0.45 | 157 | 0.65 | 5.10E-01 |
| Spleen | 0.63/0.38 | 131867 | 0.66/0.41 | 231 | 0.23 | 8.10E-01 |
| Stomach | 0.63/0.38 | 131853 | 0.79/0.52 | 245 | 1.78 | 7.00E-02 |
| Testis | 0.63/0.38 | 131603 | 0.76/0.5 | 495 | 2.04 | 4.00E-02 |
| Thyroid | 0.63/0.38 | 132004 | 0.64/0.39 | 94 | 0.96 | 3.30E-01 |
| Uterus | 0.63/0.38 | 131992 | 0.77/0.51 | 106 | 0.96 | 3.50E-01 |
| Vagina | 0.63/0.38 | 131997 | 0.67/0.42 | 101 | 0.26 | 7.90E-01 |
| Whole Blood | 0.63/0.38 | 131799 | 0.79/0.52 | 299 | 2.04 | 4.00E-02 |
| * MW(Z) is a Z statistics from Mann-Whitney test for comparing two samples. | | | | |  |  |
| All SNPs regardless of significance level were used in the analysis. | | | | |  |  |
| ** mean/median. | | | | |  |  |

**Table S3.** Mean -log_10_p for non-eQTL and eQTL backbone lung cancer OncoArray SNPs. The eQTLs are stratified by tissue types.

| eQTL tissue type | non-eQTL SNP | | eQTL SNP | | *MW(Z) | p |
| --- | --- | --- | --- | --- | --- | --- |
|  | **-log10p | N | **-log10p | N |  |  |
| Adipose Subcutaneous | 0.47/0.32 | 138217 | 0.49/0.35 | 425 | 0.31 | 7.50E-01 |
| Adipose Visceral Omentum | 0.47/0.32 | 138305 | 0.47/0.33 | 337 | 0.51 | 6.10E-01 |
| Adrenal Gland | 0.47/0.32 | 138406 | 0.49/0.35 | 236 | 0.24 | 8.10E-01 |
| Artery Aorta | 0.47/0.32 | 138293 | 0.47/0.33 | 349 | 0.33 | 7.30E-01 |
| Artery Coronary | 0.47/0.32 | 138481 | 0.43/0.29 | 161 | 1.06 | 2.80E-01 |
| Artery Tibial | 0.47/0.32 | 138201 | 0.52/0.39 | 441 | 1.53 | 1.20E-01 |
| Brain Amygdala | 0.47/0.32 | 138568 | 0.55/0.42 | 74 | 1.09 | 2.70E-01 |
| Brain Anterior cingulate cortex BA24 | 0.47/0.32 | 138481 | 0.51/0.38 | 161 | 0.7 | 4.80E-01 |
| Brain Caudate basal ganglia | 0.47/0.32 | 138444 | 0.56/0.43 | 198 | 1.92 | 6.00E-02 |
| Brain Cerebellar Hemisphere | 0.47/0.32 | 138393 | 0.51/0.38 | 249 | 0.91 | 3.50E-01 |
| Brain Cerebellum | 0.47/0.32 | 138311 | 0.5/0.36 | 331 | 0.49 | 6.20E-01 |
| Brain Cortex | 0.47/0.32 | 138413 | 0.49/0.35 | 229 | 0.1 | 9.20E-01 |
| Brain Frontal Cortex BA9 | 0.47/0.32 | 138454 | 0.53/0.4 | 188 | 1.11 | 2.70E-01 |
| Brain Hippocampus | 0.47/0.32 | 138509 | 0.54/0.41 | 133 | 1.13 | 2.50E-01 |
| Brain Hypothalamus | 0.47/0.32 | 138530 | 0.49/0.35 | 112 | 0.21 | 8.20E-01 |
| Brain Nucleus accumbens basal ganglia | 0.47/0.32 | 138446 | 0.57/0.44 | 196 | 2.03 | 4.00E-02 |
| Brain Putamen basal ganglia | 0.47/0.32 | 138491 | 0.54/0.41 | 151 | 1.24 | 2.10E-01 |
| Brain Spinal cord cervical c-1 | 0.47/0.32 | 138545 | 0.66/0.54 | 97 | 3.04 | 1.00E-03 |
| Brain Substantia nigra | 0.47/0.32 | 138561 | 0.48/0.34 | 81 | 0.03 | 9.60E-01 |
| Breast Mammary Tissue | 0.47/0.32 | 138380 | 0.43/0.29 | 262 | 0.42 | 6.70E-01 |
| Cells EBV-transformed lymphocytes | 0.47/0.32 | 138502 | 0.54/0.41 | 140 | 1.16 | 2.40E-01 |
| Cells Transformed fibroblasts | 0.47/0.32 | 138246 | 0.49/0.35 | 396 | 0.36 | 7.10E-01 |
| Colon Sigmoid | 0.47/0.32 | 138368 | 0.46/0.32 | 274 | 0.44 | 6.60E-01 |
| Colon Transverse | 0.47/0.32 | 138347 | 0.51/0.38 | 295 | 0.91 | 3.60E-01 |
| Esophagus Gastroesophageal Junction | 0.47/0.32 | 138411 | 0.47/0.33 | 231 | 0.15 | 8.70E-01 |
| Esophagus Mucosa | 0.47/0.32 | 138200 | 0.51/0.38 | 442 | 0.86 | 3.80E-01 |
| Heart Atrial Appendage | 0.47/0.32 | 138255 | 0.67/0.55 | 387 | 6.16 | 3.00E-07 |
| Heart Left Ventricle | 0.47/0.32 | 138314 | 0.52/0.39 | 328 | 1.08 | 2.70E-01 |
| Liver | 0.47/0.32 | 138463 | 0.51/0.38 | 179 | 0.63 | 5.20E-01 |
| Lung | 0.47/0.32 | 138229 | 0.49/0.35 | 413 | 0.14 | 8.80E-01 |
| Minor Salivary Gland | 0.47/0.32 | 138563 | 0.44/0.3 | 79 | 0.63 | 5.20E-01 |
| Muscle Skeletal | 0.47/0.32 | 138242 | 0.47/0.33 | 400 | 0.43 | 6.60E-01 |
| Nerve Tibial | 0.47/0.32 | 138171 | 0.49/0.35 | 471 | 0.48 | 7.70E-01 |
| Ovary | 0.47/0.32 | 138490 | 0.53/0.4 | 152 | 1.04 | 2.90E-01 |
| Pancreas | 0.47/0.32 | 138385 | 0.46/0.32 | 257 | 0.4 | 6.80E-01 |
| Pituitary | 0.47/0.32 | 138403 | 0.44/0.3 | 239 | 1.08 | 2.80E-01 |
| Prostate | 0.47/0.32 | 138478 | 0.46/0.32 | 164 | 0.57 | 5.70E-01 |
| Skin Not Sun Exposed Suprapubic | 0.47/0.32 | 138241 | 0.49/0.35 | 401 | 0.23 | 8.10E-01 |
| Skin Sun Exposed Lower leg | 0.47/0.32 | 138174 | 0.47/0.33 | 468 | 0.31 | 7.50E-01 |
| Small Intestine Terminal Ileum | 0.47/0.32 | 138484 | 0.52/0.39 | 158 | 0.86 | 3.90E-01 |
| Spleen | 0.47/0.32 | 138410 | 0.48/0.34 | 232 | 0.7 | 4.80E-01 |
| Stomach | 0.47/0.32 | 138393 | 0.46/0.32 | 249 | 0.42 | 6.70E-01 |
| Testis | 0.47/0.32 | 138140 | 0.49/0.35 | 502 | 0.5 | 6.10E-01 |
| Thyroid | 0.47/0.32 | 138154 | 0.49/0.35 | 488 | 0.45 | 6.50E-01 |
| Uterus | 0.47/0.32 | 138546 | 0.61/0.49 | 96 | 2.14 | 3.00E-02 |
| Vagina | 0.47/0.32 | 138540 | 0.44/0.3 | 102 | 0.69 | 4.90E-01 |
| Whole Blood | 0.47/0.32 | 138339 | 0.52/0.39 | 303 | 1.11 | 2.70E-01 |
| * MW(Z) is a Z statistics from Mann-Whitney test for comparing two samples. | | | | |  |  |
| All SNPs regardless of significance level were used in the analysis. | | | | |  |  |
| ** mean/median. | | | | |  |  |

**Table S4.** Mean -log_10_p for non-eQTL and eQTL scleroderma GWAS SNPs. The eQTLs are stratified by tissue types.

| eQTL tissue type | non-eQTL SNP | | eQTL SNP | | *MW(Z) | p |
| --- | --- | --- | --- | --- | --- | --- |
|  | **-log10p | N | **-log10p | N |  |  |
| Adipose Subcutaneous | 0.48/0.33 | 159962 | 0.51/0.36 | 617 | 1.01 | 3.10E-01 |
| Adipose Visceral Omentum | 0.48/0.33 | 160110 | 0.53/0.38 | 469 | 1.87 | 6.00E-02 |
| Adrenal Gland | 0.48/0.33 | 160287 | 0.53/0.38 | 292 | 1.39 | 1.60E-01 |
| Artery Aorta | 0.48/0.33 | 160103 | 0.49/0.34 | 476 | 0.31 | 7.50E-01 |
| Artery Coronary | 0.48/0.33 | 160381 | 0.52/0.37 | 198 | 0.87 | 3.80E-01 |
| Artery Tibial | 0.48/0.33 | 159974 | 0.53/0.38 | 605 | 1.83 | 7.00E-02 |
| Brain Amygdala | 0.48/0.33 | 160456 | 0.53/0.38 | 123 | 0.89 | 3.70E-01 |
| Brain Anterior cingulate cortex BA24 | 0.48/0.33 | 160393 | 0.45/0.3 | 186 | 1.03 | 3.00E-01 |
| Brain Caudate basal ganglia | 0.48/0.33 | 160289 | 0.53/0.38 | 290 | 1.41 | 1.50E-01 |
| Brain Cerebellar Hemisphere | 0.48/0.33 | 160228 | 0.49/0.34 | 351 | 0.4 | 6.80E-01 |
| Brain Cerebellum | 0.48/0.33 | 160141 | 0.51/0.36 | 438 | 0.81 | 4.10E-01 |
| Brain Cortex | 0.48/0.33 | 160249 | 0.52/0.37 | 330 | 1.09 | 2.70E-01 |
| Brain Frontal Cortex BA9 | 0.48/0.33 | 160236 | 0.51/0.36 | 343 | 0.61 | 5.40E-01 |
| Brain Hippocampus | 0.48/0.33 | 160401 | 0.46/0.31 | 178 | 0.61 | 5.40E-01 |
| Brain Hypothalamus | 0.48/0.33 | 160439 | 0.62/0.47 | 140 | 3.04 | 2.00E-04 |
| Brain Nucleus accumbens basal ganglia | 0.48/0.33 | 160347 | 0.53/0.38 | 232 | 1.19 | 2.30E-01 |
| Brain Putamen basal ganglia | 0.48/0.33 | 160370 | 0.52/0.37 | 209 | 0.86 | 3.90E-01 |
| Brain Spinal cord cervical c-1 | 0.48/0.33 | 160452 | 0.45/0.3 | 127 | 0.71 | 4.70E-01 |
| Brain Substantia nigra | 0.48/0.33 | 160497 | 0.54/0.39 | 82 | 0.87 | 3.80E-01 |
| Breast Mammary Tissue | 0.48/0.33 | 160219 | 0.54/0.39 | 360 | 2.12 | 3.00E-02 |
| Cells EBV-transformed lymphocytes | 0.48/0.33 | 160389 | 0.46/0.31 | 190 | 0.8 | 4.20E-01 |
| Cells Transformed fibroblasts | 0.48/0.33 | 160059 | 0.49/0.34 | 520 | 0.11 | 9.20E-01 |
| Colon Sigmoid | 0.48/0.33 | 160210 | 0.49/0.34 | 369 | 0.26 | 7.90E-01 |
| Colon Transverse | 0.48/0.33 | 160184 | 0.56/0.41 | 395 | 2.57 | 1.00E-02 |
| Esophagus Gastroesophageal Junction | 0.48/0.33 | 160224 | 0.55/0.4 | 355 | 2.19 | 2.00E-02 |
| Esophagus Mucosa | 0.48/0.33 | 160003 | 0.51/0.36 | 576 | 0.83 | 4.00E-01 |
| Heart Atrial Appendage | 0.48/0.33 | 160019 | 0.51/0.36 | 560 | 1.02 | 3.00E-01 |
| Heart Left Ventricle | 0.48/0.33 | 160130 | 0.53/0.38 | 449 | 1.58 | 1.10E-01 |
| Liver | 0.48/0.33 | 160207 | 0.5/0.35 | 372 | 0.29 | 7.70E-01 |
| Lung | 0.48/0.33 | 160363 | 0.49/0.34 | 216 | 0.2 | 8.40E-01 |
| Minor Salivary Gland | 0.48/0.33 | 160214 | 0.54/0.39 | 365 | 2.52 | 1.00E-02 |
| Muscle Skeletal | 0.48/0.33 | 160471 | 0.51/0.36 | 108 | 0.49 | 6.20E-01 |
| Nerve Tibial | 0.48/0.33 | 160022 | 0.5/0.35 | 557 | 0.55 | 5.80E-01 |
| Ovary | 0.48/0.33 | 159900 | 0.53/0.38 | 679 | 2.33 | 1.00E-02 |
| Pancreas | 0.48/0.33 | 160363 | 0.43/0.28 | 216 | 1.43 | 1.50E-01 |
| Pituitary | 0.48/0.33 | 160227 | 0.52/0.37 | 352 | 1.26 | 2.00E-01 |
| Prostate | 0.48/0.33 | 160263 | 0.51/0.36 | 316 | 0.8 | 4.20E-01 |
| Skin Not Sun Exposed Suprapubic | 0.48/0.33 | 160380 | 0.59/0.44 | 199 | 2.75 | 1.00E-02 |
| Skin Sun Exposed Lower leg | 0.48/0.33 | 160012 | 0.49/0.34 | 567 | 0.02 | 9.80E-01 |
| Small Intestine Terminal Ileum | 0.48/0.33 | 159923 | 0.53/0.38 | 656 | 2.11 | 3.00E-02 |
| Spleen | 0.48/0.33 | 160369 | 0.55/0.4 | 210 | 1.71 | 8.00E-02 |
| Stomach | 0.48/0.33 | 160252 | 0.51/0.36 | 327 | 0.94 | 3.40E-01 |
| Testis | 0.48/0.33 | 160289 | 0.52/0.37 | 290 | 1.18 | 2.30E-01 |
| Thyroid | 0.48/0.33 | 159881 | 0.54/0.39 | 698 | 2.39 | 1.00E-02 |
| Uterus | 0.48/0.33 | 159787 | 0.53/0.38 | 792 | 2.04 | 4.00E-02 |
| Vagina | 0.48/0.33 | 160460 | 0.44/0.29 | 119 | 0.79 | 4.20E-01 |
| Whole Blood | 0.48/0.33 | 160179 | 0.52/036 | 400 | 1.23 | 2.10E-01 |
| *MW(Z) is a Z statistics from Mann-Whitney test for comparing two samples. | | | | |  |  |
| ** mean/median. All SNPs regardless of significance level were used in the analysis. | | | | |  |  |

**Figure S1**. a) The distribution of non-overlapping 5kb fragments by the number of eQTLs. b) The distribution of 5kb non-overlapping chromosomal fragments by the number of lung GWAS SNPs. c) Distribution of 5kb non-overlapping chromosomal fragments by the number of breast cancer GWAS SNPs.


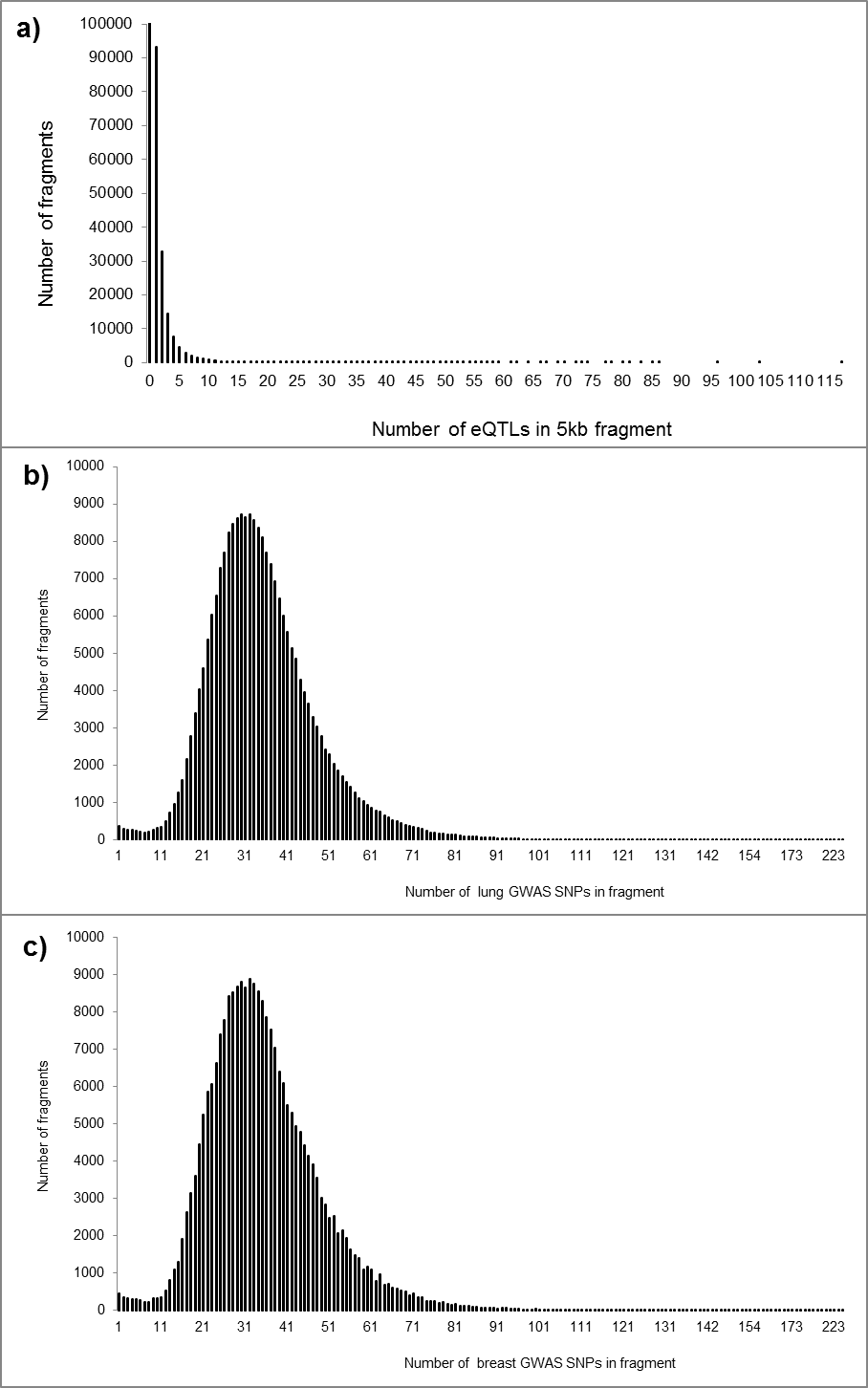


**Figure S2.** The relationship between the number of eQTLs in the ±5kb adjacent region and the level of statistical significance of the backbone SNP. **a.** Breast cancer GWAS SNPs. **b.** Lung cancer GWAS SNPs. Shaded circle indicate SNPs with >18 eQTLs in ±5kb adjacent region. Vertical bars show standard error (SE) of the mean.


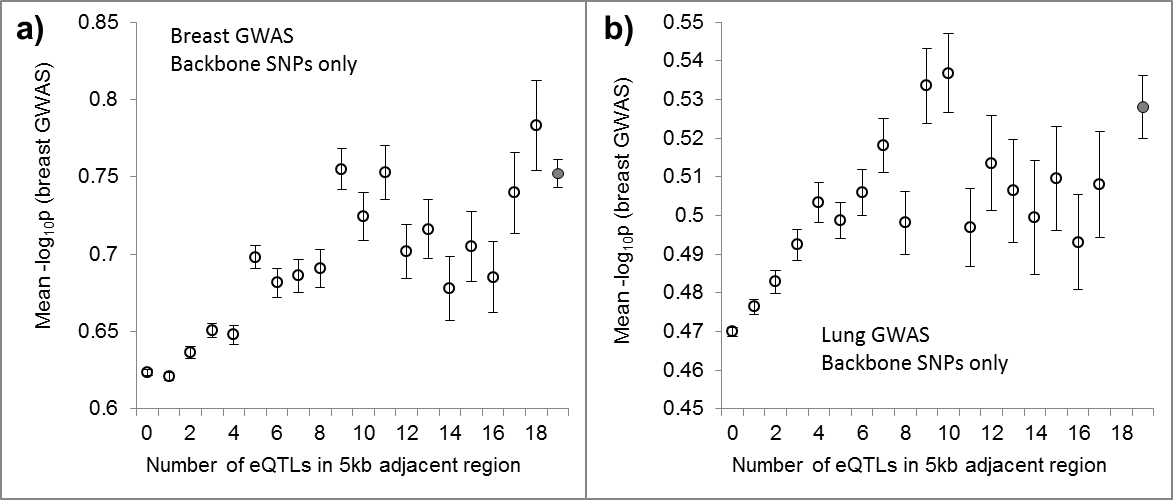


**Figure S3.** The relationship between the number of eQTLs in ±5kb adjacent region and the level of statistical significance of scleroderma SNP. Shaded circle indicate SNPs with >18 eQTLs in ±5kb adjacent region. Vertical bars show standard error (SE) of the mean.


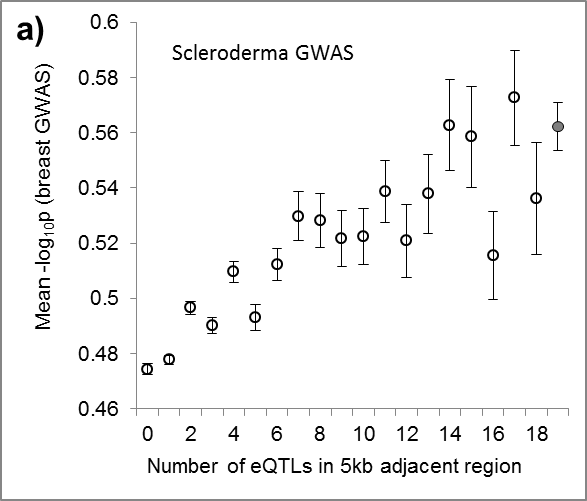

Supplement: Supplementary file 1 — Additional file 1: Table S1. Distribution of eQTL SNPs by the number of tissues where they are reported as eQTLs. eQTL SNPs with “Number of tissues” equal to one are tissue specific; others are pan-tissue. Table S2. Mean -log10p for non-eQTL and eQTL backbone breast cancer OncoArray SNPs. The eQTLs are stratified by tissue types. Table S3. Mean -log10p for non-eQTL and eQTL backbone lung cancer OncoArray SNPs. The eQTLs are stratified by tissue types. Table S4. Mean -log10p for non-eQTL and eQTL scleroderma GWAS SNPs. The eQTLs are stratified by tissue types. Figure S1. a) The distribution of non-overlapping 5 kb fragments by the number of eQTLs. b) The distribution of 5 kb non-overlapping chromosomal fragments by the number of lung GWAS SNPs. c) Distribution of 5 kb non-overlapping chromosomal fragments by the number of breast cancer GWAS SNPs. Figure S2. The relationship between the number of eQTLs in the ±5 kb adjacent region and the level of statistical significance of the backbone SNP. a. Breast cancer GWAS SNPs. b. Lung cancer GWAS SNPs. Shaded circle indicate SNPs with > 18 eQTLs in ±5 kb adjacent region. Vertical bars show standard error (SE) of the mean. Figure S3. The relationship between the number of eQTLs in ±5 kb adjacent region and the level of statistical significance of scleroderma SNP. Shaded circle indicate SNPs with > 18 eQTLs in ±5 kb adjacent region. Vertical bars show standard error (SE) of the mean. [file 12863_2019_786_MOESM1_ESM.docx]
